# Leveraging the Aggregated Protein Dye YAT2150 for Malaria Chemotherapy

**DOI:** 10.3390/pharmaceutics16101290

**Published:** 2024-09-30

**Authors:** Claudia Camarero-Hoyos, Inés Bouzón-Arnáiz, Yunuen Avalos-Padilla, Antonino Nicolò Fallica, Lucía Román-Álamo, Miriam Ramírez, Emma Portabella, Ona Cuspinera, Daniela Currea-Ayala, Marc Orozco-Quer, Maria Ribera, Inga Siden-Kiamos, Lefteris Spanos, Valentín Iglesias, Benigno Crespo, Sara Viera, David Andreu, Elena Sulleiro, Francesc Zarzuela, Nerea Urtasun, Sandra Pérez-Torras, Marçal Pastor-Anglada, Elsa M. Arce, Diego Muñoz-Torrero, Xavier Fernàndez-Busquets

**Affiliations:** 1 Barcelona Institute for Global Health (ISGlobal), Hospital Clínic-Universitat de Barcelona, Rosselló 149-153, 08036 Barcelona, Spain; claudia.camarero@isglobal.org (C.C.-H.); inesbouzonarnaiz@gmail.com (I.B.-A.); yavalos@ibecbarcelona.eu (Y.A.-P.); afallica@ibecbarcelona.eu (A.N.F.); lroman@ibecbarcelona.eu (L.R.-Á.); miriam.ramirez@isglobal.org (M.R.); portabellaemma@gmail.com (E.P.); onacuspi04@gmail.com (O.C.); daniela.currea@gmail.com (D.C.-A.); marcorozcoquer@hotmail.com (M.O.-Q.); maria.ribera@isglobal.org (M.R.); valentin.iglesias@uab.cat (V.I.); 2 Nanomalaria Group, Institute for Bioengineering of Catalonia (IBEC), The Barcelona Institute of Science and Technology, Baldiri Reixac 10-12, 08028 Barcelona, Spain; 3 Doctoral School of Biotechnology, Faculty of Pharmacy and Food Sciences, University of Barcelona, Av. Joan XXIII 27-31, 08028 Barcelona, Spain; 4 Institute of Molecular Biology and Biotechnology, FORTH, N. Plastira 100, 700 13 Heraklion, Greece; inga@imbb.forth.gr (I.S.-K.); spanos@imbb.forth.gr (L.S.); 5 Institut de Biotecnologia i de Biomedicina (IBB) and Departament de Bioquímica i Biologia Molecular, Universitat Autònoma de Barcelona, 08193 Bellaterra, Spain; 6 Clinical Research Centre, Medical University of Białystok, Kilińskiego 1, 15-369 Białystok, Poland; 7 Global Health Medicines R&D, GlaxoSmithKline (GSK), 28760 Tres Cantos, Spain; benigno.f.crespo-fernandez@gsk.com (B.C.); sara.m.viera@gsk.com (S.V.); 8 Department of Medicine and Life Sciences, Barcelona Biomedical Research Park, Pompeu Fabra University, Dr. Aiguader 88, 08003 Barcelona, Spain; david.andreu@upf.edu; 9 Microbiology Department, Vall d’Hebron University Hospital (VHUH), Universitat Autònoma de Barcelona, 08035 Barcelona, Spain; elena.sulleiro@vallhebron.cat (E.S.); francesc.zarzuela@vallhebron.cat (F.Z.); 10 Centro de Investigación Biomédica en Red de Enfermedades Infecciosas (CIBERINFEC), Carlos III Health Institute, 28029 Madrid, Spain; 11 Molecular Pharmacology and Experimental Therapeutics (MPET), Department of Biochemistry and Molecular Biology, University of Barcelona, Av. Diagonal 643, 08028 Barcelona, Spain; nurtasun@ub.edu (N.U.); s.perez-torras@ub.edu (S.P.-T.); mpastor@ub.edu (M.P.-A.); 12 Centro de Investigación Biomédica en Red de Enfermedades Hepáticas y Digestivas (CIBEREHD), Carlos III Health Institute, 28029 Madrid, Spain; 13 Institut de Recerca Hospital Sant Joan de Déu de Barcelona (IRSJD), Santa Rosa 39-57, 08950 Esplugues de Llobregat, Spain; 14 Institute of Biomedicine (IBUB), University of Barcelona, Av. Diagonal 643, 08028 Barcelona, Spain; dmunoztorrero@ub.edu; 15 Laboratory of Medicinal Chemistry (CSIC Associated Unit), Faculty of Pharmacy and Food Sciences, University of Barcelona, Av. Joan XXIII 27-31, 08028 Barcelona, Spain; e.martinezarce@ub.edu; 16 Nanoscience and Nanotechnology Institute (IN2UB), University of Barcelona, Martí i Franquès 1, 08028 Barcelona, Spain

**Keywords:** *Plasmodium falciparum*, malaria, protein aggregation, YAT2150

## Abstract

**Background/Objectives**: YAT2150 is a first-in-class antiplasmodial compound that has been recently proposed as a new interesting drug for malaria therapy. **Methods/Results**: The fluorescence of YAT2150 rapidly increases upon its entry into *Plasmodium*, a property that can be of use for the design of highly sensitive diagnostic approaches. YAT2150 blocks the in vitro development of the ookinete stage of *Plasmodium* and, when added to an infected blood meal, inhibits oocyst formation in the mosquito. Thus, the compound could possibly contribute to future transmission-blocking antimalarial strategies. Cell influx/efflux studies in Caco-2 cells suggest that YAT2150 is internalized by endocytosis and also through the OATP2B1 transporter, whereas its main export route would be via OSTα. YAT2150 has an overall favorable drug metabolism and pharmacokinetics profile, and its moderate cytotoxicity can be significantly reduced upon encapsulation in immunoliposomes, which leads to a dramatic increase in the drug selectivity index to values close to 1000. Although YAT2150 binds amyloid-forming peptides, its in vitro fluorescence emission is stronger upon association with peptides that form amorphous aggregates, suggesting that regions enriched in unstructured proteins are the preferential binding sites of the drug inside *Plasmodium* cells. The reduction of protein aggregation in the parasite after YAT2150 treatment, which has been suggested to be directly related to the drug’s mode of action, is also observed following treatment with quinoline antimalarials like chloroquine and primaquine. **Conclusions**: Altogether, the data presented here indicate that YAT2150 can represent the spearhead of a new family of compounds for malaria diagnosis and therapy due to its presumed novel mode of action based on the interaction with functional protein aggregates in the pathogen.

## 1. Introduction

The rapid emergence in *Plasmodium* of resistance to all clinically used drugs since the deployment of chloroquine in the 1940s has led to critical shortcomings in the available therapeutic and prophylactic antimalarial arsenal [1]. Parasite resistance has even appeared against the current mainstay treatment represented by artemisinin combination therapies, detected first in the Greater Mekong Subregion [2] and later in Africa [3] and South America [4]. A few new drugs are currently in the clinical pipeline [5], but all of them present significant concerns regarding resistance issues. This alarming scenario, together with the predicted climate change-driven regional expansion of the mosquito vectors of the disease and the limited resources allocated to malaria research, makes imperative the discovery of new compounds whose production is rapid and inexpensive, having new modes of action not shared by other antimalarials. This last characteristic is essential to minimize resistance evolution.

Rapid diagnostic tests (RDTs) for malaria usually rely on the antibody-mediated detection of histidine-rich protein 2, alone or in combination with other parasite antigens [6]. The antibody part of these RDTs has a short expiration time and is sensitive to the high temperature and humidity of malaria-endemic areas, thus requiring refrigerated storage. Antibodies are usually generated in hybridomas or in animals, which leads to high production costs, batch-to-batch variability, and potential ethical concerns. Because of the intracellular location of *Plasmodium* in host cells, there are few validated antigens for malaria detection, which, in addition, can disappear from the parasite’s population, leading to diagnosis failure [7,8]. Moreover, most available diagnostic strategies detect *Plasmodium falciparum*, and those developed for other species are often less sensitive [9]. The identification of *Plasmodium* antigens in blood samples is also used to confirm the elimination of the pathogen after a therapeutic treatment, although, because the decay of parasite antigens takes longer than the clearance of parasitemia, false positives are a recurrent risk [10]. In summary, improving the current diagnostic methods of malaria is an essential requirement for disease control.

**Figure 1:**
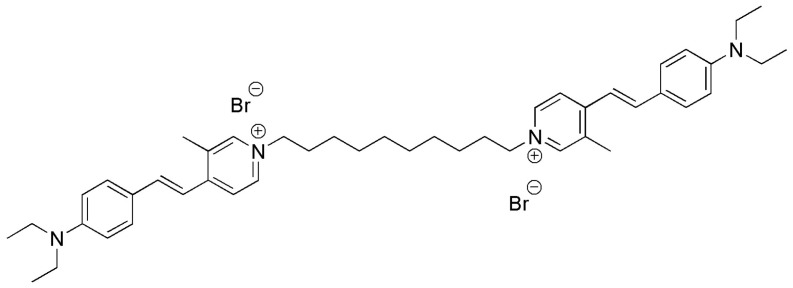
Chemical structure of YAT2150.

The active principle of the commercial ProteoStat^®^ reagent used to detect in vivo intracellular protein aggregates [11], the bis(styrylpyridinium) compound YAT2150 (Figure 1), was found to possess many of the properties that are sought after in the ideal antimalarial drug [12], namely: (i) It has an inexpensive, easy, and robust synthesis (only two steps), resulting in an excellent activity/cost ratio considering the economic landscape of the malaria endemic areas where clinical application is required; (ii) one of its presumed modes of action (protein aggregation inhibition) potentially targets a multiplicity of parasite proteins, which will likely slow down resistance evolution and might even completely prevent it; (iii) it belongs to a new chemical family in the antimalarial drug arena, which will impede the adaptation by *Plasmodium* of mechanisms of resistance to other drugs (indeed, several chloroquine- and artemisinin-resistant strains are strongly sensitive to YAT2150 [12]); (iv) it is a multi-stage compound, targeting several forms of the malaria pathogen [11,12], and is also one of the most active described compounds against *Leishmania* parasites [13]; (v) it has an in vitro half-maximal inhibitory concentration (IC_50_) below 100 nM in *P. falciparum* cultures, including gametocytes, the sexual stages responsible for *Plasmodium* transmission to mosquitoes; and (vi) it is also active against the liver stage of the parasite [12].

To progress towards the eventual entry of YAT2150 in the preclinical development phase, some of its still unknown properties have to be determined, such as the potential for diagnosis, the transmission-blocking activity, the elucidation of its cellular influx/efflux routes, and the antiparasitic mechanism of action. In addition, those known characteristics that are suboptimal must be improved. In particular, the selectivity index of YAT2150 should be increased in order to widen the therapeutic window, which can be achieved through its encapsulation in targeted nanocarriers for specific delivery to *Plasmodium*.

## 2. Materials and Methods

### 2.1. Reagents

Except where otherwise indicated, reagents were purchased from Sigma-Aldrich Corporation (St. Louis, MO, USA), and reactions were performed at room temperature (22 to 24 °C). Synthesis of YAT2150 and of the peptides KDLLF, KVVNI, and LYWIYY was conducted as previously reported [12].

### 2.2. Prediction of Prion-like Propensities

Reference proteomes from *Leishmania infantum* JPCM5 strain (proteome ID UP000008153), *Homo sapiens* (UP000005640), *Arabidopsis thaliana* (UP000006548), *Escherichia coli* (UP000000558), *Saccharomyces cerevisiae* (UP000001450), and *P. falciparum* (UP000001450), were downloaded from UniProt (release 2022_01) [14]. Proteomic prion-like propensities were calculated with PLAAC [15], where proteins reporting at least one window with COREscore > 0 were considered prion-like proteins.

### 2.3. Microscopic Observation of Clinical Samples

Clinical malaria samples were obtained from the *Centre de Salut Internacional i Malalties Transmissibles Drassanes-Vall d’Hebron*, Barcelona, Spain. A total of 5 µL of blood from a malaria-infected person were diluted in 100 µL of Roswell Park Memorial Institute 1640 medium (RPMI) supplemented with 5 mg/mL AlbuMAX^TM^ II (Invitrogen, Waltham, MA, USA) and 2 mM L-glutamine (RPMIc). The solution was stained with 2 µg/mL Hoechst 33342 and 18 µM YAT2150 and incubated for 30 min before centrifuging at 5000× *g* for 30 s and discarding the supernatant. The pellet was then resuspended in 100 µL of phosphate-buffered saline (PBS: 0.136 M NaCl, 1.47 mM KH_2_PO_4_, 8 mM NaH_2_PO_4_, and 2.68 mM KCl), and 5 µL of the suspension was placed in an 8-well slide (ibidi GmbH, Gräfelfing, Germany) with 200 µL of PBS. The sample was observed in an IX-51 Olympus (Tokyo, Japan) fluorescence microscope with a 100× objective. The fluorescence of Hoechst 33342 and YAT2150 was detected with the fluorescence filter cubes U-MNU2 and U-MWG2, which have excitation filters of 360–370 nm and 510–550 nm and emission filters of 420 nm and 590 nm, respectively.

### 2.4. Assay of Gametocyte-to-Ookinete Transition Inhibition

Eight days before ookinete production, 100 µL of a red blood cell (RBC) pellet containing the *Plasmodium berghei* circumsporozoite protein and thrombospondin-related adhesive protein (TRAP)-related protein (CTRP)-green fluorescent protein (GFP) strain (CTRP-GFP, which expresses GFP when reaching ookinete stage, kindly provided by Inga Siden-Kiamos [16]) in cryopreservation solution (RBC pellet:RPMI:30% glycerol in water, 1:1:2) was administered intraperitoneally (i.p.) to a BALB/c mouse (Charles River Laboratories, Wilmington, MA, USA). After 4 days, 5 × 10^7^ parasitized RBCs from this mouse in 100 µL of PBS were used to infect i.p. three mice that one hour before had been treated i.p. with 120 µL of a 10 mg/mL phenylhydrazine solution in PBS. For the generation of ookinetes, ca. 1 mL of blood was collected by intracardiac puncture from each animal (previously anesthetized with a 100 mg/kg ketamine plus 10 mg/kg xylazine mixture) and diluted in 30 mL of ookinete medium: 10.4 g/L RPMI, 2% *w*/*v* NaHCO_3_, 0.05% *w*/*v* hypoxanthine, 0.02% *w*/*v* xanthurenic acid, 50 U/mL penicillin and 50 µg/mL streptomycin (Invitrogen), 20% heat-inactivated fetal bovine serum (FBS, Invitrogen), 25 mM HEPES, pH 7.4. The effect of drugs on gametocyte-to-ookinete conversion was assessed in three independent replicas by plating 120 µL of each culture treated with serial dilutions of the compounds in 96-well plates and incubated for 24 h at 21 °C with orbital shaking at 50 rpm (modified from [17]). Finally, samples diluted 1:100 in PBS were analyzed in an LSRFortessa flow cytometer (BD Biosciences, Franklin Lakes, NJ, USA) set up with the 4 lasers, 20 parameters standard configuration. The GFP positive cell population was counted (λ_excitation_: 488 nm; λ_emission_: 525/40 nm) using BD FACSDiva software 6.1.3 (BD Biosciences) for data collection and Flowing Software 2.5.1 (Turku Center for Biotechnology, Turku, Finland) for analysis.

### 2.5. Membrane Blood Feeding Assay

The *Anopheles gambiae* mosquito G3 strain was reared under standard conditions at 28 °C and 80% humidity. One to two days before blood feeding, the mosquitoes were fed penicillin (20 U/mL) and streptomycin (20 μg/mL) in the sugar meal (10% sucrose). Mice were infected with the *P. berghei* mutant Bergreen, which constitutively expresses GFP [18]. Blood was obtained by heart puncture of one mouse, and aliquoted in two tubes that were immediately placed at 37 °C. YAT2150 at a final concentration of 10 µM diluted from a stock solution in dimethyl sulfoxide (DMSO) was added to one tube. The control tube contained the same concentration of DMSO (0.5%). After the feeding carried out in membrane feeders for 60 min, mosquitoes were kept at 19 °C and provided with sugar meal containing the antibiotics as above. Seven days after feeding, the mosquitoes were dissected in PBS, mounted in VECTASHIELD^®^, and GFP-positive oocysts were counted under an Axioskop 2 microscope (Zeiss, Oberkochen, Germany).

### 2.6. Cytochrome Inhibition Activity Assay

To screen the inhibition potential of YAT2150 for the recombinant human cytochrome P450 enzyme CYP3A4, the standard insect cell microsome-based protocol was followed as previously described [13], except for the use of the CYP3A4-specific inhibitor ketoconazole and substrates 7-benzyloxytrifluoromethylcoumarin (7-BFC) and dibenzylfluorescein (DBF). After 30 min of incubation, reactions were terminated by adding the corresponding stop solution (acetonitrile:0.5 M tris-HCl, 80:20 *v*/*v* for 7-BFC and 2 N NaOH for DBF).

### 2.7. Cytotoxicity Assays

THP-1 human acute monocytic leukemia cells and NCI-H460 human lung carcinoma cells were grown in RPMI supplemented with 10% FBS. Hep-G2 human hepatocellular carcinoma cells were cultured in Eagle’s minimum essential medium (EMEM) supplemented with 10% FBS. A2780 human ovarian carcinoma cells were grown in RPMI supplemented with 10% FBS and 2 mM L-glutamine. MCF-7 human breast adenocarcinoma cells were cultured in EMEM containing 2 mM L-glutamine and Earle’s balanced salt solution adjusted with 1.5 g/L sodium bicarbonate, 0.1 mM non-essential amino acids, and 1 mM sodium pyruvate and supplemented with 10% FBS. The human colorectal adenocarcinoma cell line Caco-2 (American Tissue Culture Collection, Promochem Partnership, USA) was grown in Dulbecco’s modified Eagle’s medium (DMEM) supplemented with 10% FBS and 1% penicillin/streptomycin (complete DMEM). Human umbilical vein endothelial cells (HUVEC) were grown in M199 medium supplemented with 10% FBS and 1% penicillin/streptomycin (complete M199). Except for Caco-2 and HUVEC, inhibition of cell growth was evaluated using a protocol based on monitoring the transformation into formazan of the tetrazolium salt of MTT (3-(4,5-dimethylthiazol-2-yl)-2,5-diphenyltretrazolium bromide) by metabolically active cells. Briefly, 4000 cells/well were seeded in a sterile 96-well plate and incubated for 24 h in the corresponding growth medium in an atmosphere of 95% air and 5% CO_2_ at 37 °C. Then, a serial dilution of YAT2150 was added from a stock solution in DMSO. After 96 further h of incubation in the same conditions, 10 µL of a 5 mg/mL MTT solution in PBS was added to each sample, and the incubation was resumed for another 4 h. Then, 100 µL of a 10% sodium dodecyl sulfate solution in 0.01 M HCl was added. The incubation was continued for an additional 12 to 14 h, and absorbance was measured at 595 nm (Tecan M1000 Infinite^®^ Pro, Tecan Trading AG, Männedorf, Switzerland). The absorbance measurement range was framed between one sample containing 4000 cells in plain growth medium (to determine the stable cell concentration) and another sample containing an equal cell number in growth medium supplemented with growth factors (to measure the maximum cell growth at 96 h). For Caco-2 cells and HUVEC (100,000 and 50,000 cells/mL, respectively), plates containing 100 µL/well were incubated for 48 h, when the medium was replaced by 100 µL of, respectively, complete DMEM or complete M199 containing 0.00125% of resazurin sodium salt. Cells were incubated for another 5 h, and resorufin fluorescence emission was measured in a Tecan Infinite 200 PRO equipment (Tecan Trading AG) using excitation and emission wavelengths of 535 and 590 nm, respectively. Controls containing DMSO at the same concentration present in all YAT2150 dilutions were included in the assay. These controls showed an inhibition of cell growth of 6 to 8% relative to the control in which the cells were grown in plain growth medium. All the experiments were performed three times in triplicate and the results are reported as the YAT2150 concentration required for the reduction of cell viability by 50% (CC_50_) ± standard error of the mean (SEM). CC_50_ values were calculated using the nonlinear regression fit analysis in GraphPad Prism 8.4 (GraphPad software, La Jolla, CA, USA), whereas SEM values were determined with Microsoft Office Excel version 2401.

### 2.8. Human Plasma Protein Binding Determination

The assay was carried out by Rapid Equilibrium Dialysis (RED). The compounds were dissolved at 5 μM in human plasma and added to the corresponding insert of the RED device (Thermo Fisher Scientific, Inc., Waltham, MA, USA), and dialysis was performed for 4 h at 37 °C in 0.15 mM NaCl, 25 mM tris-HCl, pH 7.2 (Tris BupH^TM^, Thermo Fisher Scientific, Inc.). Then, 50-µL aliquots from each chamber were transferred to empty vials, to which 50 µL of dialysis buffer was added. As control, 50 µL of plasma was mixed with 50 µL of buffer. Three hundred µL of acetonitrile was added to all the samples, which were analyzed by ultra-performance liquid chromatography with tandem mass spectrometry as previously described [13].

### 2.9. Influx and Efflux Assays in Caco-2 Cells

The panel of influx and efflux membrane transporters expressed in Caco-2 cells has been previously characterized and, to a great extent, mimics the one described in human intestine [19,20,21,22,23,24]. Based upon this information and the established knowledge of the physiology of intestinal transporters, we considered the following as possible YAT2150 influx transporters: Organic Cation Transporters (OCTs), Peptide Transporter 1 (PEPT1), and Organic Anion Transporter Polypeptide 2B1 (OATP2B1). Candidates to mediate YAT2150 efflux were Multidrug Resistance Protein 2 (MRP2), Breast Cancer Resistance Protein (BCRP), Multidrug Resistance Protein 1 (MDR1), also known as P-glycoprotein (P-gp), Organic Solute Transporter α (OSTα), and Equilibrative Nucleoside Transporters 1 and 2 (ENT1-2). For all of them, there are suitable inhibitors (see below). The role of endocytosis in YAT2150 accumulation in Caco-2 cells can also be tackled using inhibitors of the various types of endocytic mechanisms.

Caco-2 cells were grown in monolayer on a solid support at 37 °C in a humidified atmosphere with 5% CO_2_ and maintained in DMEM supplemented with 10% FBS, 2 mM L-glutamine, 1% MEM non-essential amino acids, 20 U/mL penicillin, and 20 µg/mL streptomycin (Life Technologies, Carlsbad, CA, USA). To identify YAT2150 influx and efflux mechanisms in Caco-2 cells, YAT2150 intracellular accumulation was determined by measuring its fluorescent emission at 610 nm under various experimental conditions as follows. A total of 50,000 cells/well were seeded in MW24 plates, and 24 h after seeding, YAT2150 influx and efflux were determined either in the presence or absence of FBS. For influx measurements, Caco-2 cells were preincubated for 20 min with various influx inhibitors, and they were further incubated with 5 µM YAT2150, in the presence of inhibitors, for 30 min. Then, cells were washed with PBS, and intracellular YAT2150 was determined (see below). For efflux measurements, cells were incubated with 5 µM YAT2150 alone or combined with the different efflux inhibitors for 30 min, washed with PBS, and incubated with the inhibitors alone, and intracellular YAT2150 was measured 4 h later. Intracellular fluorescent YAT2150 was detected by fluorescence microscopy (Thunder Imaging System, Leica Microsystems GmbH, Wetzlar, Germany) and analyzed by ImageJ 1.53q software (National Institutes of Health, Bethesda, MD, USA). In parallel, emitted fluorescence was measured in an iBright Imaging System and quantified by iBright Analysis Software 5.3.0 (Thermo Fisher Scientific, Inc.). Fluorescence in cells cultured for the same time in the absence of YAT2150 provided the negative control of the assay. YAT2150 fluorescence in cells cultured in the absence of inhibitors was the reference condition to which all data in the presence of inhibitors (and the negative control too) was compared. Inhibitors and concentrations used for influx transporters were 100 µM quinine (OCTs inhibitor), 100 µM losartan (PEPT1 inhibitor), and 100 µM rifampin (OATP2B1 inhibitor). Inhibitors and concentrations used for efflux transporters were 1 mM probenecid (MRP2 inhibitor), 10 µM Ko143 (BCRP inhibitor), 10 µM elacridar (P-gp inhibitor), 200 µM spironolactone (OSTα inhibitor), and 10 µM dipyridamole (ENTs inhibitor). These concentrations were chosen based on previously published transporter inhibitory profiles of these molecules (Supplementary Table S1). Endocytosis was blocked using either 20 µM dynasore (dynamin inhibitor), 20 µg/mL pitstop2 (clathrin inhibitor), or 100 µg/mL nystatin (caveolin inhibitor). Quinine, losartan, dynasore, pitstop2, nystatin, probenecid, Ko143, spironolactone, and dipyridamole were from Merck KGaA (Darmstadt, Germany) and rifampin and elacridar from Selleck Chemicals LLC (Houston, TX, USA). Data are expressed as mean ± SEM. Statistical significance was determined using Student’s *t*-test with Excel. Differences were considered significant when *p* < 0.05.

### 2.10. Treatment of P. falciparum Cultures

The asexual blood stages of *P. falciparum* parasites of the 3D7 strain (Malaria Research and Reference Reagent Resource Center, Manassas, VA, USA) were cultured in group B human RBCs at 3% hematocrit, which was kept throughout the experiments, and incubated in RPMIc. Parasites were maintained at 37 °C under an atmosphere of 5% O_2_, 5% CO_2_, and 90% N_2_. Growth inhibition assays were performed as reported elsewhere [12]. Briefly, serial dilutions of the compounds either free in solution or encapsulated in liposomes [13] or in immunoliposomes targeted to red blood cells with antibodies against glycophorin A [25] were prepared in RPMIc in triplicates and mixed with ring stage parasites (synchronized by 5% sorbitol treatment as previously described [26]) to a final volume of 300 µL and 1.5% parasitemia in 96-well plates. A positive growth control, consisting of untreated parasites, and a negative growth control, where parasites were exposed to a lethal dose of chloroquine (1 µM), were included. The parasites were then cultured under standard conditions for 48 h, allowing for a complete replication cycle. After the incubation period, 3 µL of culture from each well was mixed with 197 µL of PBS containing 0.1 µM Syto 11 (Thermo Fisher Scientific, Inc.) to obtain a final concentration of 1 × 10^6^ to 10 × 10^6^ cells/mL. Parasitemia levels were measured using an LSRFortessa flow cytometer equipped with a standard configuration of four lasers and 20 parameters. Single-cell populations were identified on a forward-side scatter plot. The fluorescence signal from Syto 11 was detected by exciting the samples at 488 nm and collecting the emission with a 530/30 nm bandpass filter. Growth inhibition was calculated by comparing the growth rates of untreated cultures and those treated with chloroquine. The inhibition data were then transformed using sigmoidal fitting to determine IC_50_.

For stage arrest assays, *P. falciparum* cultures were tightly synchronized in schizont stages by repeated treatment with 70% Percoll (GE Healthcare, Chicago, IL, USA) [26,27]. Half of each culture remained untreated, and the other half was treated with the IC_80_ of YAT2150. At different time points up to 72 h, culture samples were stained with Giemsa, and the number of rings, early and mature trophozoites, and schizonts for each time point was determined by microscopic examination of at least 100 parasitized RBCs for each sample, counted in 10 fields/slide from microscope slides prepared from two independent experiments performed in triplicate. Pictures were taken with a Nikon Eclipse 50i microscope equipped with a DS-Fi1 camera (Nikon Corporation, Tokyo, Japan).

To determine the parasite-killing profile [28], 3D7 *P. falciparum* cultures were treated for 24 and 48 h with ten times the in vitro IC_50_ of the tested drugs (a concentration that had been shown to avoid suboptimal drug exposure [29]), with medium refreshed daily. After the treatment, the drug was removed and the culture diluted 1/3 and re-established in RBCs labeled with carboxyfluorescein diacetate succinimidyl ester (CFDA-SE, Life Technologies) to a 2% hematocrit in order to evaluate the reinfection capacity of the treated parasites. CFDA-SE-labelled erythrocytes were prepared by incubating 1% hematocrit in RPMI containing 10 μM CFDA-SE at 37 °C for 30 min, after which they were washed and maintained at 50% hematocrit at 4 °C for up to 24 h before use. Parasites were grown for 48 h, a complete replication cycle, in standard culturing conditions (5% O_2_, 5% CO_2_, and 90% N_2_ at 37 °C). After this incubation time, parasitemia was assessed by two-color flow cytometry (Attune NxT Flow Cytometer, Thermo Fisher Scientific, Inc.) using Hoechst 33342 to stain nuclei. Hoechst 33342 fluorescence signal was detected by exciting samples at 405 nm and collecting the emission with a 440/50-nm bandpass filter (VL1), whereas CFDA-SE was excited at 488 nm and detected with a 530/30 nm filter. Samples were analyzed using the Attune NxT software package 3.2.1, and parasite viability was plotted as the percentage of infected CFDA-SE-stained erythrocytes, using as reference untreated samples of the initial inoculum after 48 h of incubation with labeled erythrocytes.

### 2.11. In Vitro Peptide Aggregation Assays

Stock solutions of the peptides LYWIYY, KDLLF, and KVVNI were prepared in DMSO (without previous peptide disaggregation) and diluted 1:100 or 1:1000 in PBS to final concentrations of 545.7, 225, and 562.5 µM, respectively. Samples were vortexed, and complete aggregation was stimulated by incubation at 37 °C under continuous stirring at 1400 rpm in a ThermoMixer^®^ (Eppendorf, Hamburg, Germany) for 48 h. Then, peptides were further diluted in triplicates to 12.5 µM in a 96-well black plate (Greiner Bio-One, Madrid, Spain) in a final volume of 100 µL of PBS/well and stirred for 1 h at 37 °C. For thioflavin T (ThT) fluorescence measurements, ThT was added to a final concentration of 25 µM from a PBS stock, and samples were stirred again for 15 min at 37 °C in the dark. Fluorescence was collected from 460 to 800 nm using an Infinite Nano+ multimode microplate reader (Tecan Trading AG) using an excitation wavelength of 450 nm. For YAT2150 fluorescence measurements, YAT2150 was added at a final concentration of 10 µM from a DMSO stock, and fluorescence was immediately collected between 495 and 850 nm using an excitation wavelength of 485 nm. Results were plotted by normalization of obtained fluorescence intensity values *vs* the highest ThT and YAT2150 fluorescence intensities experimentally registered. For all samples, the final DMSO content was kept below 1% to minimize interferences with the aggregation process and control spectra of YAT2150 and ThT in PBS were subtracted from those obtained in the presence of peptides.

### 2.12. Transmission Electron Microscopy

To disaggregate peptides before the assay, 1 mg of lyophilized peptide was dissolved in 1 mL of trifluoroacetic acid (TFA), which, after thoroughly mixing, was evaporated under a N_2_ stream, and 500 µL of 1,1,1,3,3,3-hexafluoro-2-propanol (HFIP; Honeywell Fluka-Thermo Fisher Scientific, Inc.) were added, mixed well, and evaporated as mentioned before (repeated twice to fully remove TFA). Then, 500 µL of HFIP was added, and the solution was divided into aliquots and placed in a desiccator overnight. Afterward, 500 µL of PBS was added to each aliquot to have a final peptide concentration of 25 µM, and, to ensure minimal aggregation (t_0_), the samples were sonicated for 10 min in a water bath sonicator (FB15053 ultrasonic bath, Thermo Fisher Scientific, Inc.). To study the effect of YAT2150 on peptide aggregation, the compound was added at a final concentration of 100 nM and incubated for 24 h at 37 °C under stirring at 1400 rpm (t_24_). For disaggregation analysis, a 25 µM peptide aliquot was first pre-aggregated for 24 h (37 °C, 1400 rpm); after this time, YAT2150 was added at a final concentration of 100 nM, and the samples were incubated for an additional 24 h in the same conditions (t_48_). Control samples in the absence of YAT2150 were included. A 5-μL drop of each sample was deposited onto a carbon-coated copper grid (Ted Pella, Redding, CA, USA) for 1 h. Next, the grid was washed on top of a 20 µL double deionized water drop for 5 min. Subsequently, it was deposited onto a 20 µL drop of 2% uranyl acetate and allowed to stain for 2 min. The resulting samples were observed with a JEM 1010 transmission electron microscope (JEOL Ltd., Tokyo, Japan), and images were acquired using an Orius 832 CCD camera (Gatan, Inc., Pleasanton, CA, USA).

### 2.13. Protein Aggregation Assays in P. falciparum Cultures

A 3D7 *P. falciparum* culture enriched in schizont stages by 70% Percoll density centrifugation (1070× *g*, 10 min) was treated for 4 h at 37 °C with the IC_50_ of artemisinin, atovaquone, chloroquine, and primaquine (10.8 nM, 1 nM, 7 nM, and 3 µM, respectively, as determined in our experimental setting). After this incubation, 9 mL of culture was removed and subjected to a second Percoll treatment to pellet the parasitized red blood cells, which were resuspended in 50 μL of lysis buffer, consisting of 4.5 mg/mL NaCl in H_2_O supplemented with 1× cOmplete^TM^ protease inhibitor cocktail (Roche, Basel, Switzerland), and treated as described elsewhere [13]. The rest of the culture was incubated in the presence of the drugs’ IC_50_ for a further 48 h, when parasitemia was determined by optical microscopic counting of Giemsa-stained slides and compared to that of an untreated control culture. Only those samples with parasitemias between 30 and 70% relative to the control were considered for ThT analysis.

### 2.14. In Vivo Toxicity Determination

The *Caenorhabditis elegans* N2 Bristol strain (kindly provided by Dr. Christian Griñán-Ferré) was cultured, following established methods [30], in 90 mm Petri dishes containing solid Nematode Growth Media (NGM) seeded with *E. coli* OP50 (100 µL of an overnight culture spread on top of the NGM), maintained at 20 °C, and expanded to new plates once a week by transferring a 1-cm^3^ NGM cube to a new plate. *C. elegans* synchronization at the first larval stage was achieved with bleaching solution (75% H_2_O, 20% commercial bleach, 5% 10 M NaOH), selectively allowing survival of only eggs. To do this, a plate with gravid adult worms was washed twice with 5 mL of M9 buffer (20 mM KH_2_PO_4_, 42 mM Na_2_HPO_4_, 86 mM NaCl, 1 mM MgSO_4_), and the nematodes were transferred to a 15-mL conical tube, where they were pelleted (400× *g*, 2 min). Two washes were then performed with 10 mL of M9, and the resulting *C. elegans* pellet was suspended in 5 mL of bleaching solution and gently mixed for 9 min, when the reaction was stopped by adding 5 mL of M9. Then the eggs were pelleted (400× *g*, 1 min), washed 3× with M9, and finally suspended in 200 µL of M9 and added to a fresh solid NGM plate without *E. coli*. A total of 12 to 24 h after bleaching synchronization, the plate was washed with 10 mL of M9 to collect the worms, which were pelleted (400× *g*, 2 min), taken up in M9 supplemented with *E. coli* (100 µL of an overnight bacterial culture/mL M9), and distributed in several wells of a 96-well plate to have 30 to 40 worms/well, to which were added serial dilutions of YAT2150 in solution or encapsulated into liposomes in a final volume of 200 µL/well and incubated at 20 °C. *C. elegans* viability and body length were analyzed after 48 h under a stereomicroscope (Leica M50 Routine Stereo Microscope; Leica Microsystems GmbH), taking representative images and videos with a 4× objective. *C. elegans* viability was determined in three experimentally independent replicas according to two variables: body shape (stiff, rod-like worms were counted as dead, as opposite to s-shaped live worms) and presence of movement in living *C. elegans*, with an n ≥ 100 worms/sample. Nematode body length was calculated with ImageJ software in worms anesthetized with 50 mM sodium azide. Statistically significant differences between groups were determined by the two-way analysis of variance (ANOVA) using GraphPad Prism 9.0.1.

### 2.15. Ethical Issues

The human blood used for *P. falciparum* cultures was commercially obtained from the *Banc de Sang i Teixits* (www.bancsang.net). Blood was not specifically collected for this research; the purchased units had been discarded for transfusion, usually because of an excess of blood relative to anticoagulant solution. Prior to their use, blood units underwent the analytical checks specified in the current legislation. Before being delivered to us, unit data were anonymized and irreversibly dissociated, and any identification tag or label had been removed in order to guarantee the non-identification of the donor. The human biological samples to perform the parasite viability assay were sourced ethically, and their research use was in accordance with the terms of the informed consent. GSK acknowledges the *Centro de Hemoterapia y Donación de Valladolid, Castilla y León*, and the *Centro de Transfusiones de la Comunidad de Madrid* for the supply of blood samples. No personal data were or will be supplied in accordance with the current Spanish *Ley Orgánica de Protección de Datos* and *Ley de Investigación Biomédica*. The blood samples will not be used for studies other than those made explicit in this research.

Six-week-old BALB/c mice (around 20 g in weight) were kept in specific pathogen-free animal research facilities in ventilated racks with ad libitum access to food and water, with 12/12 h light/dark cycles, and were acclimatized for one week after arrival. In the presence of toxic effects including, among others, > 20% reduction in weight, aggressive and unexpected animal behavior, or the presence of blood in feces, mice were immediately anesthetized using a 100 mg/kg ketamine plus 10 mg/kg xylazine mixture and sacrificed by cervical dislocation. The animal care and use protocols followed adhered to the specific national and international guidelines in accordance with the current Catalan (D 214/1997/GC) and Spanish laws (RD 53/2013; order ECC/566/2015), Greek law (2015/92 and PD 56/2013) and the corresponding European Directive (2010/63/EU). The studies reported here involving mice were performed under protocols reviewed and approved by the Ethics Committee on Drug Research from the *Hospital Clínic de Barcelona* (www.clinicbarcelona.org/ceim, accessed on 10 June 2021; Reg. HCB/2021/1258, 17 February 2022) and by FORTH Ethics Committee and by the Prefecture of Crete (license number 106323, 29 April 2021). Clinical sample collection was approved by the Ethics Committee from the *Institut de Recerca Vall d’Hebron*, register number PR(AG) 68/2020.

### 2.16. Statistical Data Analysis

The results are expressed as mean values ± SEM unless otherwise indicated. Significance was established at *p* < 0.05 for the corresponding tests indicated in the table and figure legends. Non-linear regression analysis was used to determine cytotoxicity and drug activity values. Oocyst numbers were analyzed by a non-parametric Mann–Whitney test.

## 3. Results

### 3.1. Rapid Detection of Malaria Infection in YAT2150-Stained Clinical Samples

**Figure 2:**
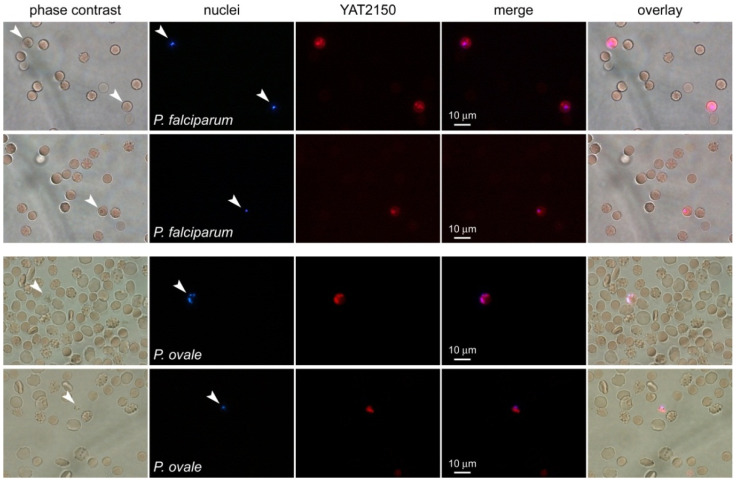
YAT2150 staining of clinical samples of *P. falciparum* and *P. ovale* infections. The merge panels refer to fluorescence images only. Arrowheads indicate the *Plasmodium*-infected red blood cells present in the microscope fields shown.

YAT2150 strongly fluoresces when in contact with aggregated protein regions in the malaria parasite [11,12], a property that can be instrumental for the design of new diagnostic approaches. In clinical samples, YAT2150 allowed for the easy identification of *P. falciparum*- and *Plasmodium ovale*-parasitized red blood cells (pRBCs), which otherwise were much harder to detect by relying on their weak nuclear staining (Figure 2).

### 3.2. Transmission Blocking Assays

**Figure 3:**
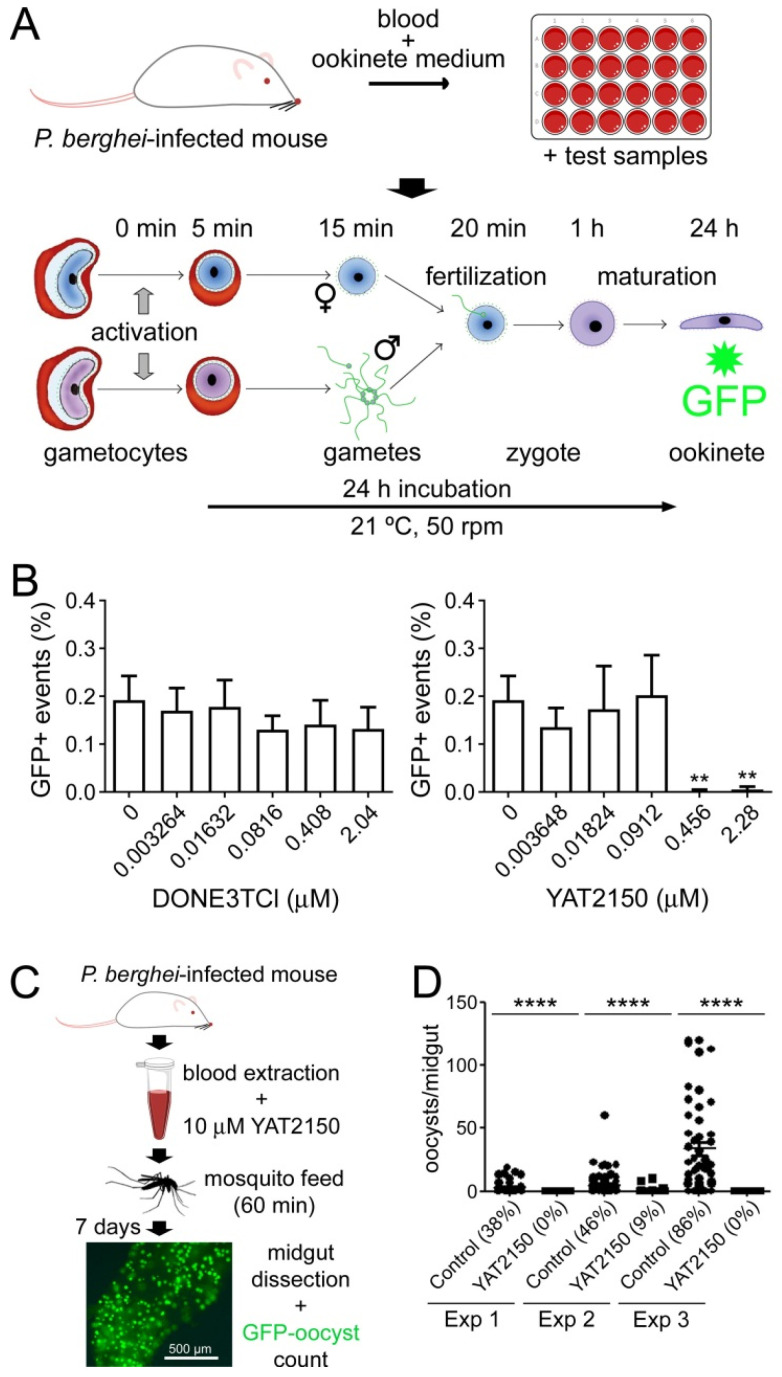
Transmission blocking assays. (**A**,**B**) Ex vivo *P. berghei* ookinete maturation assay. (**A**) Scheme of the experimental protocol. (**B**) Effect of DONE3TCl and YAT2150 on ookinete development. Mean values ± standard deviations are indicated (n = 3). **: *p* < 0.01 (one-way ANOVA, Dunnett’s post-hoc test). (**C**,**D**) Membrane feeding assay to test the effect of YAT2150 on *P. berghei* oocyst production. (**C**) Scheme of the experimental protocol. A total of 10 µM YAT2150 was added to blood infected with *P. berghei* and immediately fed to *A. gambiae* mosquitoes. Control feeding was the same blood mixed with DMSO at the same concentration as in the YAT2150-containing sample. The illustrative GFP-oocyst image is from Lantero et al. [33]. (**D**) Effect of YAT2150 on oocyst development in three independent experiments (Exp 1 to 3). Percentages in the graph indicate the prevalence of infection (% of infected mosquitoes). ****: *p* < 0.0001 (Mann–Whitney non-parametric test).

In a typical malaria infection, a fraction of the circulating pRBCs differentiate into male and female gametocytes, which are responsible for the transmission of *Plasmodium* to the mosquito vector of the disease. In the insect’s midgut, gamete fertilization leads to the formation of a motile zygote termed ookinete, which crosses the midgut epithelium to form an oocyst where sporozoites, the parasite stage that infects humans, develop. Thus, transmission-blocking drugs are one of the mainstays of malaria control strategies. The in vitro IC_50_s of YAT2150 for *P. falciparum* stage I-III and stage V gametocytes were, respectively, 95 and 103 nM [12], significantly lower than that of the reference gametocytocidal drug primaquine (around 20 µM for stage IV-V gametocytes [31,32]). To further study this potent activity on gametocytes, the effect of YAT2150 on one of the key steps of the pathogen’s development in the mosquito, namely the gametocyte-to-ookinete transition, was tested. Ex vivo ookinete maturation assays in the murine malaria parasite *P. berghei* (Figure 3A) indicated that ca. 0.5 µM YAT2150 abolished ookinete production in this model (Figure 3B). In contrast, the aminoquinoline DONE3TCl (belonging to a chemical family that includes the quinoline antimalarial drugs), which showed potent antiplasmodial activity in in vitro *P. falciparum* asexual blood stage cultures, with an IC_50_ of ca. 80 nM [12], had a much less significant effect on ookinete development up to a concentration of 2 µM.

In order to test whether YAT2150 also had an effect on the development of the parasite in the mosquito, preliminary membrane-feeding assays were carried out (Figure 3C). *A. gambiae* mosquitoes were offered blood meals of *P. berghei*-infected blood to which a final concentration of 10 µM YAT2150 was added immediately before feeding. This relatively high concentration was chosen to account for the observation that mosquitoes start excreting drops of fluid one minute after feeding begins [34], which would likely remove most of the ingested YAT2150 after a very short time. Oocysts counted after 7 days revealed that both the prevalence of infection and oocyst number/midgut were significantly reduced in the mosquitoes fed on blood containing YAT2150 compared to the control (Figure 3D).

### 3.3. Drug Metabolism and Pharmacokinetics (DMPK) and Early Safety Profiling of YAT2150

The good profile of YAT2150 as a prospective antimalarial drug warranted a consistent characterization of its physicochemical, DMPK, and early safety properties. Recently, we reported a preliminary assessment of some of them [13], which is completed here with additional assays (Table 1). The YAT2150 concentration required for the reduction of cell viability by 50% (CC_50_) in seven different human cell lines ranged between 0.59 and 18.2 µM depending on the cell type. The sensitivity to YAT2150 of tumoral cells was found to be generally significantly higher than to the reference anticancer agent cisplatin (Supplementary Table S2). Inhibition assays of cytochrome P450 (CYP) enzymes indicated an IC_50_ of 1.0, 1.1, and 1.7 µM for CYP3A4, CYP2C19, and CYP2D6 isoforms, respectively, whereas, at 10 µM YAT2150, the inhibition of CYP1A2 and CYP2C9 isoforms was around 50%. YAT2150 was less active than the reference cytochrome inhibitors, except for CYP2C19, where YAT2150’s IC_50_ was similar to that of tranylcypromine (Supplementary Table S2). The stability at 37 °C in human plasma of YAT2150 was high, with 91.3% of it remaining after 6 h. Upon incubation with human microsomes at 37 °C, 55% of YAT2150 remained unchanged after 1 h, with a half-life of 80.5 min and an intrinsic clearance (Clint) of 10.6 µL/min/mg protein, which represented higher stability than that of the reference compound testosterone (Supplementary Table S2). YAT2150 stability was also analyzed in human hepatocytes to find a Clint of 2.5 µL/min/10^6^ cells, similar to that of prazosin and better than that of imipramine (Supplementary Table S2). The human plasma protein binding of YAT2150 was 99.7%.

### 3.4. Cell Influx and Efflux Studies

**Figure 4:**
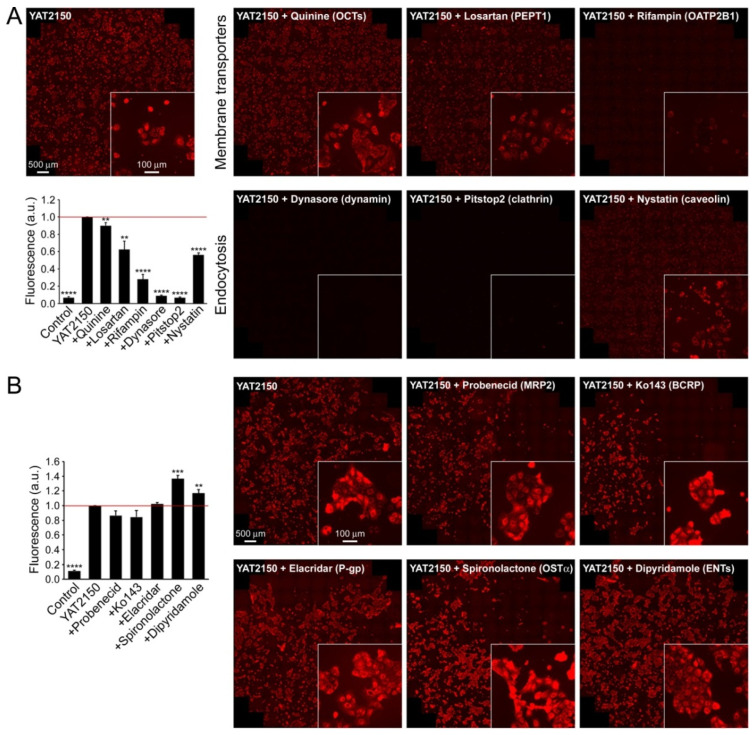
YAT2150 (**A**) influx and (**B**) efflux study in Caco-2 cells in FBS-free medium. Representative microscopy images of YAT2150 fluorescence from three to five independent experiments are shown. Each panel corresponds to a single inhibitor blocking a particular pathway (in parenthesis). The bar graphs show the quantification of YAT2150 accumulation as reported in Materials and Methods using a fluorescence imaging system and normalized to YAT2150 without inhibitors (YAT2150, red line). The negative control sample (control) corresponds to the fluorescence in cells cultured for the same time in the absence of YAT2150 (no image of this condition is provided because fluorescence was almost negligible). Results are the mean ± SEM from three to five independent experiments. Statistical significance relative to YAT2150 influx and efflux was determined by Student’s *t*-test: ** *p* < 0.01, *** *p* < 0.001, **** p < 0.0001.

Bidirectional permeability assays in a Caco-2 cell monolayer (Table 1), commonly used to predict the in vivo intestinal absorption of drugs, indicated an elevated absorption (>200 nm/s) and moderate efflux (ca. 100 nm/s) for YAT2150 [13], which resulted in a favorable efflux ratio of 0.5, significantly better than for common reference compounds such as colchicine and estrone-3-sulfate, with efflux ratios of 33 and 26 nm/s, respectively (Supplementary Table S2). Influx studies with suitable inhibitors in Caco-2 cells in an FBS-free medium showed that YAT2150 was mainly internalized by endocytosis and also through the OATP2B1 transporter (Figure 4A). Experiments aiming at identifying efflux routes showed that OSTα/β transporters mediated YAT2150 efflux (Figure 4B). Qualitatively, the same results were obtained in a medium containing 10% FBS (Supplementary Figures S1 and S2), although in this case, the intracellular YAT2150-associated fluorescence was significantly lower, probably due to its strong association with plasma proteins indicated by DMPK data (Table 1). Other transporters and efflux pumps expressed in Caco-2 cells, such as OCTs, PEPT1, ENTs, MRP2, BCRP, and P-gp, did not appear to contribute significantly to transmembrane YAT2150 translocation.

Although RBCs do not endocyte, they express OATP2B1 [35], suggesting that this transporter might be an import route of YAT2150 into erythrocytes. Once YAT2150 is internalized in Caco-2, it is mainly retained in cellular structures, likely protein-enriched regions as it has been shown to occur in *P. falciparum*-infected erythrocytes [12]. The fact that non-parasitized RBCs are not stained by YAT2150 (Figure 2) indicates either that the drug does not enter naïve erythrocytes in significant amounts through the OATP2B1 transporter or that it does not emit fluorescence inside these cells because they lack aggregated proteins, as indicated by previous ThT fluorescence analysis of non-parasitized RBCs [12]. The affinity of YAT2150 for lipid bilayers [13] suggests an alternative physicochemical way of going in and out of cells based on an initial insertion into the plasma membrane followed by its cell entry or exit according to the corresponding intra- and extracellular protein compositions.

### 3.5. In Vitro Antiplasmodial Activity of YAT2150 Encapsulated in Liposomes

**Figure 5:**
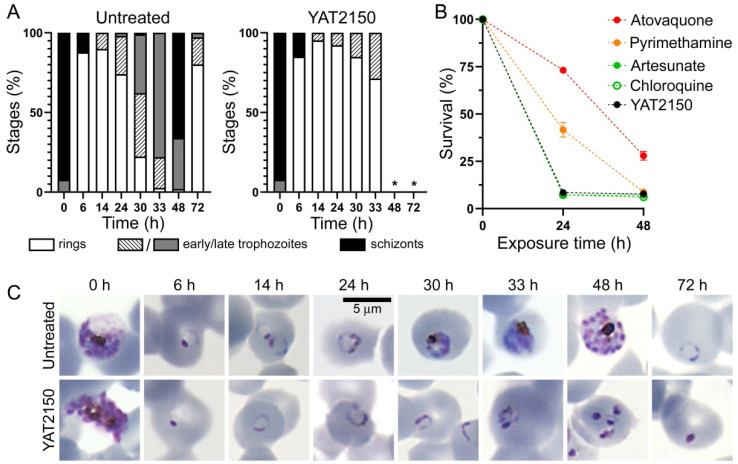
Characterization of the fast-acting activity of YAT2150. (**A**) Stage arrest assay of 3D7 *P. falciparum* synchronized at schizont stage, treated for up to 72 h with the IC_80_ of YAT2150 (200 nM). At the indicated times after treatment start, blood smears stained with Giemsa were prepared, and parasite population was noted for at least 100 pRBCs. Bars show the percentages of asexual blood stages present at each time; *: indicates the presence of only pyknotic and dead parasites of which the population could not be annotated (n = 2 independent experiments). (**B**) Parasite-killing profile of 3D7 *P. falciparum* parasites treated for 24 and 48 h with 10 times the IC_50_ of YAT2150 or with fast- (in green, chloroquine and artesunate), moderate- (in orange, pyrimethamine), and slow-acting (in red, atovaquone) antimalarials. (**C**) Representative images of Giemsa-stained pRBCs in the stage arrest assay of panel (**A**).

YAT2150 is a fast-acting antiplasmodial according to stage arrest assays performed in *P. falciparum* ring stages [12] and schizonts (Figure 5A,C), when the drug arrested the parasite’s growth in the next phase (trophozoites and rings, respectively). These results were confirmed in parasite reduction ratio assays (Figure 5B), which showed an 8.5% parasite survival after treatment for 24 h with 10× IC_50_, a growth inhibition similar to that induced by the fast-acting antimalarial drugs artesunate and chloroquine that were used as controls, in parallel to the slower-acting antimalarials atovaquone and pyrimethamine.

The relatively high cytotoxicity of YAT2150 (Table 1) could be significantly reduced upon encapsulation of the drug in liposomes and immunoliposomes [13]. When YAT2150 was incorporated into *P. falciparum* in vitro cultures for only two hours (to mimic its estimated progressive removal from the blood circulation in an eventual in vivo assay) its IC_50_ was 224 ± 27 nM, similar to when an equal amount of the compound was added encapsulated in liposomes (Table 2). However, the encapsulation of YAT2150 in immunoliposomes targeted with antibodies against the red blood cell surface protein glycophorin A (GPA) (Supplementary Figure S3), resulted in a dramatic drop in IC_50_ to 51 ± 3 nM after only two hours of incubation with the parasite. This low IC_50_ combined with the reduced cytotoxicity of YAT2150-loaded liposomes compared to the free compound led to a selectivity index close to 1000 units for the drug encapsulated in anti-GPA immunoliposomes. The in vivo toxicity of YAT2150 in the *C. elegans* model (half-maximal inhibitory concentration of 16.2 ± 1.4 µM for the free drug, Table 1) was also significantly reduced to >50 µM following its encapsulation in liposomes (Supplementary Figure S4). Because of the incorporation in the liposome lipid bilayer of YAT2150 [13], which imposes a limit on its maximum load, 50 µM is the highest concentration of the compound that could be reliably tested in such a nanocarrier targeted to glycophorin A because higher immunoliposome amounts can lead to agglutination.

### 3.6. Effect of Clinically Used Antimalarial Drugs on the Level of Protein Aggregation in P. falciparum Cultures

**Figure 6:**
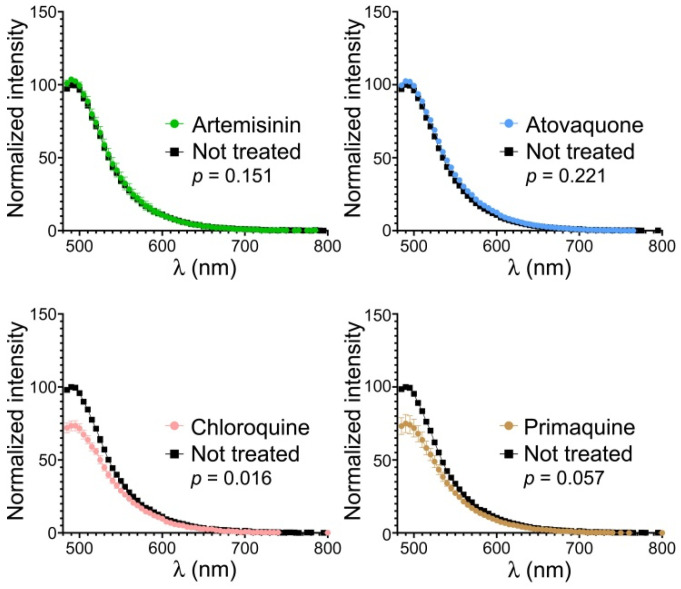
ThT analysis in *P. falciparum* asexual blood stage cultures of the effect on protein aggregation of four clinically used antimalarial drugs. ThT fluorescence assay of *P. falciparum* culture extracts normalized to have equal protein content, either non-treated or treated for 4 h with artemisinin, atovaquone, chloroquine, or primaquine at their respective in vitro IC_50_ (10.8 nM, 1 nM, 7 nM, and 3 µM, respectively, as determined in our experimental setting). The *p*-values refer to the fluorescence intensity measured at the maximum emission wavelength.

The presumed antiplasmodial mode of action of YAT2150 has been proposed to be the inhibition of protein aggregation in the parasite [12], although the precise underlying mechanism has not been characterized yet. Certain small molecules that contained the quinoline scaffold characteristic of some widely used antimalarial drugs like chloroquine and primaquine had been described to inhibit in vitro the aggregation of several amyloid peptides [36]. When tested in in vitro cultures of *P. falciparum*, these compounds exhibited the expected antiplasmodial activity derived from their quinoline nature [37], a result that represented the first empirical indication of a connection between peptide/protein aggregation inhibition and antimalarial activity. When chloroquine and primaquine were subjected to the ThT assay for the determination of their effect on the aggregative state of the *P. falciparum* proteome in in vitro cultures of the parasite [12], they were found to reduce protein aggregation in the parasites (Figure 6). In contrast, non-quinoline antimalarials like artemisinin and atovaquone did not have an observable effect on the aggregation of the pathogen’s proteome.

### 3.7. Interaction of YAT2150 with Aggregative Peptides

**Figure 7:**
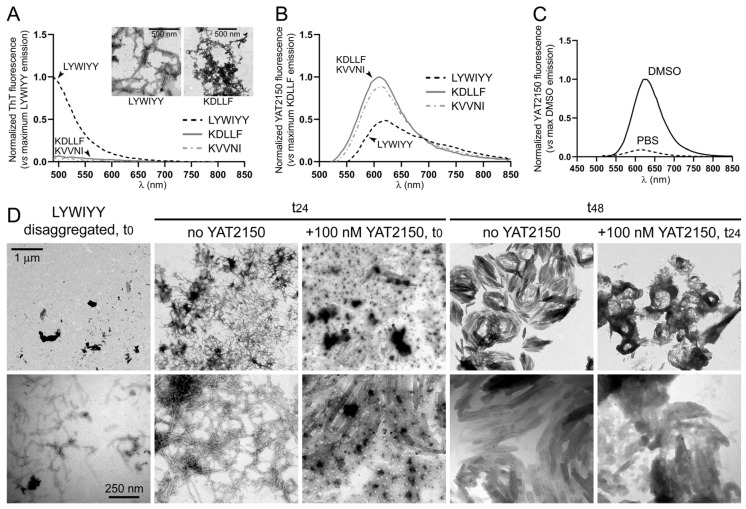
Interaction of YAT2150 with aggregative peptides. (**A**–**C**) Relative fluorescence emission intensity of solutions of non-disaggregated KDLLF, KVVNI, and LYWIYY peptides treated with YAT2150 and ThT. Peptides (12.5 µM each) were incubated in the presence of (**A**) 25 µM ThT or (**B**) 10 µM YAT2150 before proceeding to measuring fluorescence emission. The insets in panel (**A**) show representative TEM images of the aggregates formed by LYWIYY and KDLLF. (**C**) Fluorescence emission spectra of peptide-free 10 µM YAT2150 solutions in PBS (dashed line) and DMSO (solid line). (**D**) Transmission electron microscopy analysis of the effect of 100 nM YAT2150 on the aggregation of LYWIYY at 24 h and 48 h after undergoing a disaggregation process. Size bars: 1 µm (upper panels, 30,000×), 250 nm (lower panels, 120,000×).

YAT2150 binds protein aggregates in live cells [12], but the nature of these aggregates is unknown. As a first approximation to investigate this, three peptides present in *P. falciparum* proteins, which had been previously tested for their different aggregation propensities [12], were used: LYWIYY, which formed typical amyloid fibrils, and KDLLF and KVVNI, which mainly formed amorphous unstructured aggregates. The fluorescence of the amyloid dye ThT was >10-fold more intense when exposed to LYWIYY than in the presence of KDLLF or KVVNI (Figure 7A), in agreement with the different amyloid nature of the peptides. However, the relative fluorescence intensities were reversed upon exposure of the peptides to YAT2150, being in this case the signal for KDLLF and KVVNI significantly stronger than for LYWIYY (Figure 7B). This result suggested that YAT2150 is preferentially interacting with non-amyloid protein aggregates present inside *P. falciparum*. Remarkably, the fluorescence of YAT2150 increased significantly (ca. 10-fold) when dissolved in DMSO instead of PBS (Figure 7C). This observation is in agreement with the hypothesis that YAT2150 has a high affinity for interactions with lipophilic environments, such as those of organic solvents like DMSO, lipid bilayers [13], or the hydrophobic milieu encountered inside peptide and protein aggregates.

YAT2150 has been described to inhibit the aggregation of the model amyloidogenic peptide Aβ fragment 1–40 [12]. To investigate this activity with an aggregative peptide present in *P. falciparum*, we performed a transmission electron microscopy analysis of the effect of physiological YAT2150 concentrations close to the drug’s in vitro IC_50_ on the aggregation of LYWIYY (Figure 7D), which, according to ThT assays, had a strong amyloid nature (Figure 7A). Following a disaggregation procedure to bring the peptide solution close to the start of its aggregation dynamics, after 24 h of incubation the sample was almost exclusively composed of amyloid fibrils, which after a further 24 h evolved to structures that tended to assemble in circular shapes. The presence of 100 nM YAT2150 throughout the incubation time since disaggregation significantly abolished after 24 h the formation of LYWIYY amyloid fibrils, and, when added to fibrils (after 24 h of incubation in the absence of the drug), it clearly impaired their evolution towards the nest-like structures observed after 48 h of incubation in the control sample without YAT2150.

## 4. Discussion

In a malaria infection, the mature *Plasmodium* asexual blood stages adhere to the microvasculature capillaries, and, therefore, the only circulating form of the pathogen that can be used for diagnosis in a blood sample is the ring stage, which is a pRBC containing a single *Plasmodium* cell. The detection of ring forms requires highly specialized microscopists to identify the infection accurately in, e.g., a Giemsa-stained slide, with sufficient rapidity to decide on adequate treatment. The strong YAT2150 fluorescence observed, shortly after the compound’s addition, in circulating ring stages of *P. falciparum* and *P. ovale* clinical samples offers good prospects for the development of fast sensitive diagnostic tests that are essential for making rapid decisions on the correct treatment to be administered to malaria patients.

The inhibitory activity of YAT2150 on gametocyte maturation and ookinete development makes it a promising drug candidate for transmission-blocking approaches and for the implementation of eventual antimalarial strategies contemplating the delivery of antiplasmodials to the mosquito vector [38]. The in vitro IC_50_ of YAT2150 for *P. falciparum* gametocyte cultures (ca. 100 nM [12]) is much lower than that reported for the reference gametocytocidal antimalarial drug in clinical use, primaquine (around 20 µM [31,32]). Although primaquine significantly increases its in vivo antimalarial activity after being metabolized in the liver [39], in vitro data obtained in the human hepatoma cell line Huh7 showed that YAT2150 is about 10 times more potent than primaquine against liver stages of the murine malaria parasite *P. berghei* (respective IC_50_s of 0.78 µM [12] and ca. 10 µM [40]). Liver metabolism of primaquine has as a consequence a high increase in oxidative stress [39], which limits the use of the drug, especially for patients deficient in glucose-6-phosphate dehydrogenase [41]. On the contrary, the high activity of YAT2150 against gametocytes does not require its passage through the liver.

The cell permeability exhibited by YAT2150 can be exploited for new therapeutic strategies such as proteolysis-targeting chimeras (PROTACs), bifunctional molecules that artificially enhance the removal of a protein by recruiting cellular components that facilitate its elimination [42]. Usually, a drug binding a target parasite protein is conjugated to a moiety designed to recruit an E3 ligase that brings into proximity the target with molecular elements that will ubiquitylate and mark it for proteasomal degradation. However, although *Plasmodium* has several E3 ligases, future work will require their characterization, the design of adequate ligands for them, and of the PROTAC molecules themselves [43].

The cytotoxicity of YAT2150 varies among different cell types by more than one order of magnitude, with some tumoral cell lines being particularly sensitive to it. Recently, YAT2150 has been shown to rapidly reduce ATP levels in the leishmaniasis parasite *Leishmania major* [13], suggesting that more metabolically active cells might have a higher susceptibility to the drug. Since glycolysis is the main pathway for ATP production in *P. falciparum* [44] and in tumoral cells [45], it can be speculated that some of the molecular targets of YAT2150 might be glycolysis-related proteins, although further experimentation will be required to investigate this possibility in more detail. The encapsulation in targeted nanocarriers for increased specific delivery to the parasite will additionally afford longer blood circulating times and protection of the compound from degradation [46]. Liposomes and anti-GPA immunoliposomes have been used here as a proof-of-concept to evaluate the improvement in the therapeutic window of YAT2150 through both increasing its activity against the parasite and reducing its toxicity for the host’s cells. The data obtained will be highly informative regarding future work addressing the encapsulation of YAT2150 in other types of targeted polymeric nanocarriers that have shown better potential than liposomes for oral administration formulations [47,48,49,50].

Previous analyses had revealed an astonishing one-fourth of the *P. falciparum* proteome having prion-like domains (PrLDs), which, after adjusting for the parasite’s biological background, was later lowered to ca. 10% of its proteins [51]. This represents one of the highest proportions of predicted proteins with characteristics of prions among all the organisms where this phenomenon has been studied [52] (Table 3). *P. falciparum* PrLDs were shown to have amyloid cores capable of forming amyloid fibrils [51], and the existence in live *P. falciparum* blood stages of abundant aggregative proteins has been empirically demonstrated [11,12], whereby protein aggregation has been suggested to be functional for the pathogen [12].

Quinolines like chloroquine and primaquine have been shown to reduce protein aggregation in in vitro *P. falciparum* cultures according to ThT assays. Other quinolines that inhibit *P. falciparum* growth [12] have been described to be inhibitors of the aggregation of different amyloidogenic peptides [36], suggesting that the antimalarial activity of quinoline compounds might have a component related to the interference with protein aggregation, in addition to their well-known main mechanism of action consisting on the arrest of heme group detoxification through the inhibition of hemozoin crystal formation [53]. Actually, several clinically used quinoline antimalarial drugs, like quinine, chloroquine, primaquine, amodiaquine, quinacrine, and mefloquine, reduce the aggregation of prion proteins, and other antimalarials, such as methylene blue, curcumin, and quercetin, have been described to prevent amyloid β peptide aggregation, whereas the antiplasmodial macrocyclic lactone rapamycin decreased in vivo protein aggregation (reviewed in [12]). This accumulated evidence strongly suggests that the limitation of protein aggregation in *Plasmodium* might be one of the mechanisms behind the mode of action of many antimalarial drugs, in agreement with the proposed hypothesis that some molecular targets of antiprion and antimalarial substances overlap [54]. The interference of YAT2150 with the aggregation dynamics of amyloid peptides and its preferential binding to unstructured peptide aggregates suggests that the affinity of YAT2150 for disordered protein regions might upset critical molecular interactions. This, in turn, could affect downstream events that are essential for cell survival and thus might be the basis for the antiplasmodial mechanism of this compound.

## 5. Conclusions

YAT2150 is a fast-acting antiplasmodial compound that allows rapid fluorescence-based identification of *Plasmodium*-infected red blood cells. YAT2150 is internalized by endocytosis and also through the OATP2B1 transporter, and it blocks the in vitro development of the ookinete stage of *Plasmodium*. YAT2150 has an overall favorable drug metabolism and pharmacokinetics profile and its encapsulation in glycophorin A-targeted immunoliposomes leads to a selectivity index > 900. Unstructured protein regions are the preferential binding sites of this compound in *Plasmodium*.

## Figures and Tables

**Table 1:** DMPK and early safety profiling of YAT2150.

IC_50_ in *P. falciparum* asexual blood stages (nM)		90.5 ± 7.0
IC_50_ in *P. falciparum* stage I–III/stage V gametocytes (nM)		95 ± 2/103 ± 2 ^1^
In vivo toxicity in *C. elegans* (IC_50_, µM)		16.2 ± 1.4
HUVEC inhibition (CC_50_, µM)		14.8 ± 3.8
Caco-2 inhibition (CC_50_, µM)		18.2 ± 2.6
MCF7 ^2^ inhibition (CC_50_, µM)		0.93 ± 0.01
NCI-H460 ^3^ inhibition (CC_50_, µM)		6.76 ± 0.27
THP-1 ^4^ inhibition (CC_50_, µM)		0.66 ± 0.01
HEP-G2 ^5^ inhibition (CC_50_, µM)		2.54 ± 0.08
A2780 ^6^ inhibition (CC_50_, µM)		0.59 ± 0.16
CYP1A2 inhibition (% at 10 µM)		55 ± 1 ^7^
CYP2C9 inhibition (% at 10 µM)		51 ± 4 ^7^
CYP2C19 inhibition (IC_50_, µM)		1.1 ^7^
CYP2D6 inhibition (IC_50_, µM)		1.7 ^7^
CYP3A4 inhibition (7-BFC ^8^, % at 10 µM/DBF ^9^, IC_50_, µM)		35 ± 3/1.0
Solubility in PBS (µM)		8.5 ^7^
Human plasma protein binding (%)		99.7
Stability in human plasma	(% remaining after 1 h)	100.0 ^7^
	(% remaining after 2 h)	99.7 ^7^
	(% remaining after 6 h)	91.3 ^7^
Microsomal stability	(% remaining after 1 h)	54.6 ^7^
	T_1/2_ (min)	80.5 ^7^
	Clint (µL/min·mg prot)	10.6 ^7^
Hepatocyte stability	(% remaining after 2 h)	70.4 ^7^
	T_1/2_ (min)	277.3 ^7^
	Clint (µL/min·10^6^ cells)	2.5 ^7^
Transport through Caco-2	AB (Papp, nm/s)	207.6 ± 11.6 ^7^
	BA (Papp, nm/s)	100.6 ± 0.2 ^7^
	Efflux ratio	0.48 ± 0.03 ^7^

^1^ From [12]. ^2^ Human breast adenocarcinoma. ^3^ Human lung carcinoma. ^4^ Human acute monocytic leukemia. ^5^ Human hepatocellular carcinoma. ^6^ Human ovarian carcinoma. ^7^ From [13]. ^8^ 7-benzyloxytrifluoromethylcoumarin (7-BFC) as substrate. ^9^ Dibenzylfluorescein (DBF) as substrate.

**Table 2:** Selectivity index determination in *P. falciparum* blood stages of YAT2150 either in free form or encapsulated in liposomes and anti-GPA immunoliposomes.

	IC_50_ ± SEM (nM) ^1^	CC_50_ ± SEM (µM) ^2^	SI (CC_50_/IC_50_)
Free YAT2150	224 ± 27	14.8 ± 3.8	~66
YAT2150-liposomes	218 ± 8	>50 ^3^	>229
YAT2150-immunoliposomes	51 ± 3	>50 ^3^	>980

^1^ Two-hour incubation. ^2^ In HUVEC. ^3^ From [13].

**Table 3:** Prediction of prion-like propensities in different organisms.

Species	Taxonomic Lineage	Number of Proteins	Predicted Prion-Like Proteins (^1^)
*Plasmodium falciparum*	Eukaryota—Protist	5375	704 (13.1%)
*Leishmania infantum*	Eukaryota—Protist	8044	127 (1.6%)
*Homo sapiens*	Eukaryota—Animalia	79,052	982 (1.2%)
*Arabidopsis thaliana*	Eukaryota—Plantae	39,328	752 (1.9%)
*Saccharomyces cerevisiae*	Eukaryota—Fungi	6062	267 (4.4%)
*Escherichia coli*	Prokaryota—Eubacteria	5061	7 (0.1%)

^1^ Percentage of the total number of proteins.

## Data Availability

All the data supporting the reported results can be found in the main article and in the Supplementary Materials files.

## Supplementary Materials

The following are available online at https://www.mdpi.com/article/10.3390/pharmaceutics16101290/s1, Supplementary Table S1: Inhibitors used to determine YAT2150 influx and efflux mechanisms. Supplementary Table S2: DMPK and early safety properties of YAT2150, including reference compounds. Supplementary Figure S1: YAT2150 influx study in Caco-2 cells in medium containing 10% FBS. Supplementary Figure S2: YAT2150 efflux study in Caco-2 cells in medium containing 10% FBS. Supplementary Figure S3: Immunoliposome characterization. Supplementary Figure S4: YAT2150 in vivo toxicity assay in *C. elegans*.
