# Supplementary material for: Leveraging the Aggregated Protein Dye YAT2150 for Malaria Chemotherapy
Source: Pharmaceutics. 2024 Sep 30;16(10):1290. doi: 10.3390/pharmaceutics16101290 (PMC11514582; doi:10.3390/pharmaceutics16101290)
Supplement: Supplementary file 1 [file pharmaceutics-16-01290-s001.zip › pharmaceutics-3110476-supplementary.pdf]

Supplementary Materials

Leveraging the Aggregated Protein Dye YAT2150 for Malaria Chemotherapy

Supplementary Tables and Figures

Supplementary Table S1. Inhibitors used to determine YAT2150 influx and efflux mechanisms.

	Inhibitor	Concentration used	Transporter	References
Influx	Quinine	100 µM	OCTs	[1]
	Losartan	100 µM	PEPT1	[2]
	Rifampin	100 µM	OATP2B1	[3]
Efflux	Probenecid	1 mM	MRP2	[4]
	Ko143	10 µM	BCRP	[5]
	Elacridar	10 µM	P-gp	[6]
	Spironolactone	200 µM	OSTα	[7,8]
	Dipyridamole	10 µM	ENT1-2	[9]

Supplementary Table S2. DMPK and early safety properties of YAT2150, including reference compounds tested in parallel. In bold font are reported data extracted from the references indicated in the footnote.

		YAT2150	Reference compound
IC_{50} *P. falciparum* (3D7) blood stages		90.5±7.0 nM	**29.7±6.0 nM**[1]
IC_{50} *P. falciparum* early/stage V gametocytes		95±2 nM/103±2 nM	**18.9±2.0 μM**[2]
CC_{50} HUVEC		14.8±3.8 μM	**2.35±0.04 μM**[3]
CC_{50} Caco-2		18.2±2.6 μM	**107 μM**[4]
CC_{50} MCF7 human breast adenocarcinoma		0.93±0.01 μM****	14.30±1.20 μM[5]
CC_{50} NCI-H460 human lung carcinoma		6.76±0.27 μM***	3.69±0.29 μM[6]
CC_{50} THP-1 human acute monocytic leukemia		0.66±0.01 μM^{ns}	1.87±0.03 μM[7]
CC_{50} HEP-G2 human hepatocellular carcinoma		2.54±0.08 μM****	9.20±0.27 μM[8]
CC_{50} A2780 human ovarian carcinoma		0.59±0.16 μM^{ns}	0.87±0.14 μM[9]
CYP1A2 inhibition (at 10 μM)		55±1%	61% (at 2 μM)[10]
CYP2C9 inhibition (at 10 μM)		51±4%	43% (at 0.37 μM)[11]
CYP2C19 inhibition (IC_{50})		1.1 μM	43% (at 3.3 μM)[12]
CYP2D6 inhibition (IC_{50})		1.7 μM	69% (at 7.3 nM)[13]
CYP3A4 inhibition (7-BFC, at 10 μM/DBF, IC_{50})		35±3%/1.0 μM	48% (0.027 μM)[14]
Solubility in PBS		8.5 μM	8.0 μM[15], 63.8 μM[16]
Human plasma protein binding		99.7%	83.3%[17]
Microsomal stability	(remaining after 1 h)	54.6%	6.5%[18]
	$T_{1/2}$ (min)	80.5	15.3[18]
	CLint (μl/min/mg prot)	10.60	55.65[18]
Hepatocyte stability	(remaining after 2 h)	70.4%	70.2%[19], 30.0%[20]
	$T_{1/2}$ (min)	277.3	241.5[19], 79.3[20]
	CLint (μl/min/10^6 cells)	2.5	2.9[19], 8.7[20]
Transport through Caco-2	AB (Papp, nm/s)	207.6±11.6****, ****	2.6±0.2[21], 4.4±0.3[22]
	BA (Papp, nm/s)	100.6±0.2$^{ns, ns}$	89.8±4.7[21], 110.0±9.4[22]
	Efflux ratio	0.48±0.03***, **	35.3±3.0[21], 26.5±3.5[22]

[1]Chloroquine [10].
[2]Primaquine [10].
[3]Cisplatin [11].
[4]Cisplatin [12].
[5]Cisplatin (CC_{50} of 16.7 μM reported [13]).
[6]Cisplatin (CC_{50} of 2 μM reported [14]).
[7]Cisplatin (CC_{50} of 2 μM reported [15]).
[8]Cisplatin (CC_{50} of 15 μM reported [16]).
[9]Cisplatin (reported CC_{50} of 0.16 μM [17], 1.2 μM [18], and 2.3 μM [19]).
[10]Furafylline (IC_{50} of 5.2 μM reported [20]).
[11]Sulfaphenazole (IC_{50} of 0.64 μM reported [20]).
[12]Tranylcypromine (IC_{50} of 3.2 μM reported [21]).
[13]Quinidine (IC_{50} of 4 nM reported [20]).
[14]Ketoconazole with BFC as substrate (IC_{50} of 0.01 μM reported [20]).
[15]Progesterone (3.7 μM reported [22]).
[16]Prazosin (26.3 μM reported [22]).
[17]Phenytoin.
[18]Testosterone.
[19]Prazosin (CLint of 2.3 μl/min/10^6 cells reported [23]).
[20]Imipramine (CLint of 8.0 μl/min/10^6 cells reported [23]).
[21]Colchicine (Papp AB/BA of 13/54 nm/s reported [24]).
[22]Estrone-3-sulfate (Papp AB/BA of 27/175 nm/s reported [25]).
**: $p < 0.01$; ***: $p < 0.001$; ****: $p < 0.0001$; ^{ns}: not significative (two-way ANOVA, Dunnett's post-hoc test).

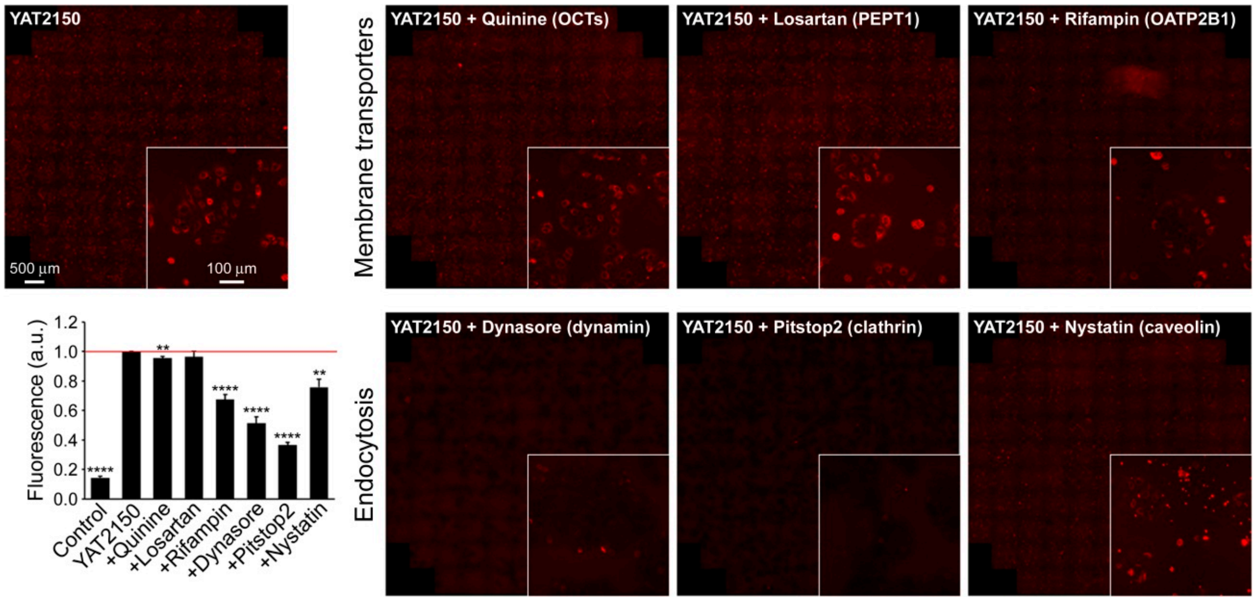

Supplementary Figure S1. YAT2150 influx study in Caco-2 cells in medium containing 10% FBS. After a 20-min pre-incubation with several inhibitors of influx mechanisms (transporters and endocytosis pathways), Caco-2 cells were further incubated with 5 μM YAT2150 for 30 min in the presence of the inhibitors. Representative microscopy images of YAT2150 fluorescence from three to four independent experiments are shown. Each panel corresponds to a single inhibitor blocking a particular influx pathway (in parenthesis). The bar graph shows the quantification of YAT2150 accumulation as explained in Materials and Methods using a fluorescence imaging system and normalized to YAT2150 without inhibitors (YAT2150, red line). Results are the mean ± SEM of three or four independent experiments. Statistical significance relative to YAT2150 influx was determined by Student's t-test: ** $p < 0.01$, **** $p < 0.0001$.

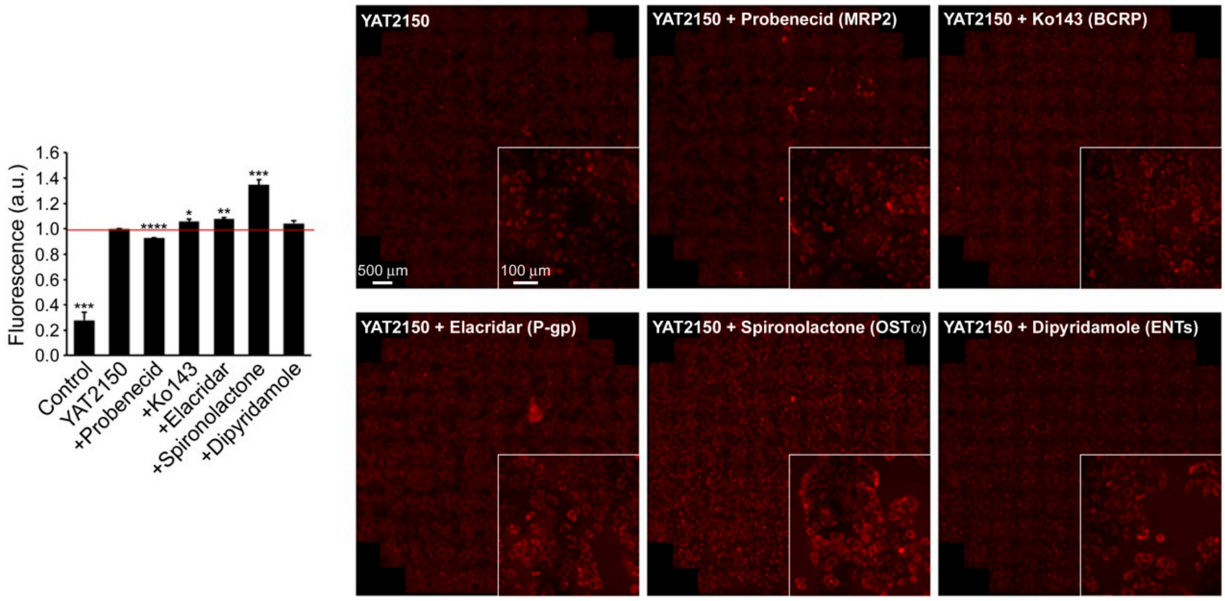

Supplementary Figure S2. YAT2150 efflux study in Caco-2 cells in medium containing 10% FBS. Caco-2 cells were incubated with YAT2150 and efflux inhibitors for 30 min, and then washed with PBS and incubated with efflux inhibitors alone. YAT2150 intracellular accumulation was determined 4 h later. Representative microscopy images of YAT2150 fluorescence from three independent experiments are shown. Each panel corresponds to a single inhibitor blocking a particular efflux pathway (in parenthesis). The bar graph shows the quantification of YAT2150 accumulation as explained in Materials and Methods using a fluorescence imaging system and normalized to YAT2150 without inhibitors (YAT2150, red line). Results are the mean ± SEM of three independent experiments. Statistical significance relative to YAT2150 efflux was determined by Student's t-test: * $p < 0.05$, ** $p < 0.01$, *** $p < 0.001$, **** $p < 0.0001$.

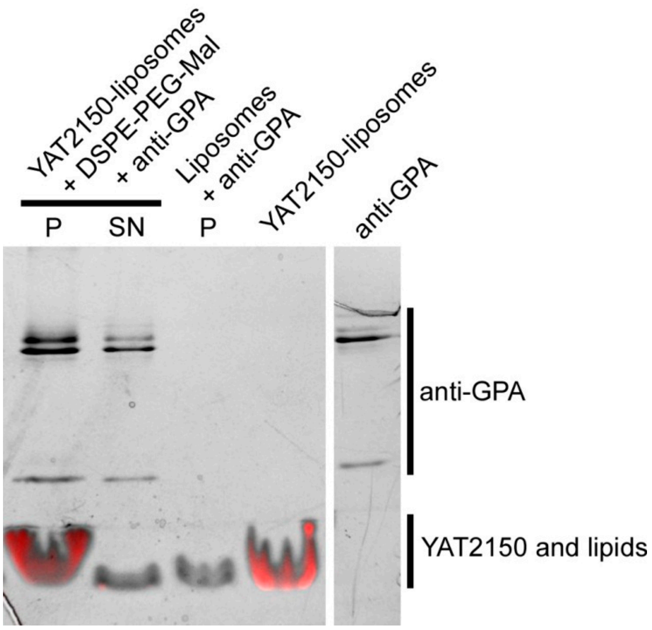

Supplementary Figure S3. Immunoliposome characterization (adapted from [26]). Silver-stained SDS-PAGE analysis of YAT2150-containing immunoliposomes functionalized with anti-glycophorin A (GPA) monoclonal antibody, compared to control liposomes lacking DSPE-PEG-Mal and subjected to the same experimental procedure. SN: supernatant after ultracentrifugation, P: pellet after ultracentrifugation, taken up in the same volume as the supernatant of PBS containing 10 mM EDTA.

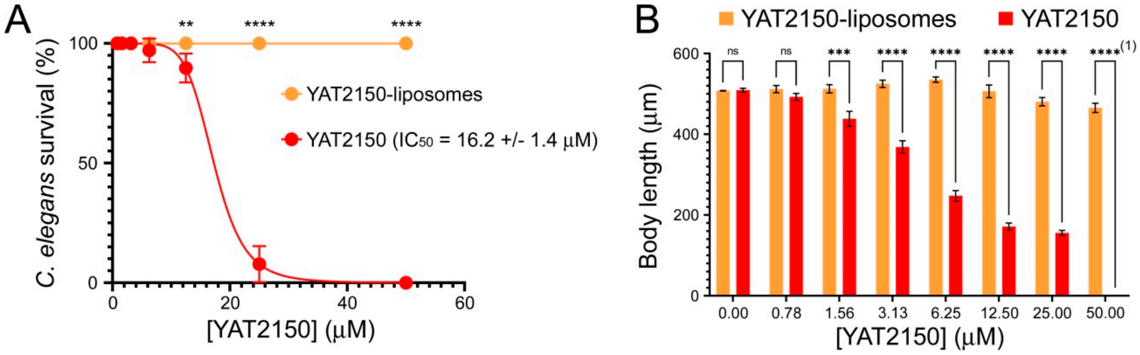

Supplementary Figure S4. YAT2150 *in vivo* toxicity assay in *C. elegans*. (**A**) Nematode survival assay. (**B**) Nematode length assay. ** $p < 0.01$, *** $p < 0.001$, **** $p < 0.0001$; ns: not significant. [(1)] No worms could be found in the non-encapsulated 50 µM YAT2150 sample.

References

1. Koepsell, H. Organic cation transporters in health and disease. *Pharmacol. Rev.* **2020,** *72* (1), 253-319.
2. Knütter, I.; Kottra, G.; Fischer, W.; Daniel, H.; Brandsch, M. High-affinity interaction of sartans with H^+/peptide transporters. *Drug Metab. Dispos.* **2009,** *37* (1), 143-149.
3. Choi, M. K.; Jin, Q. R.; Choi, Y. L.; Ahn, S. H.; Bae, M. A.; Song, I. S. Inhibitory effects of ketoconazole and rifampin on OAT1 and OATP1B1 transport activities: considerations on drug-drug interactions. *Biopharm. Drug Dispos.* **2011,** *32* (3), 175-184.
4. Namkoong, E. M.; Kim, I. W.; Kim, D. D.; Chung, S. J.; Shim, C. K. Effect of probenecid on the biliary excretion of belotecan. *Arch. Pharm. Res.* **2007,** *30* (11), 1482-1488.
5. Zechner, M.; Castro Jaramillo, C. A.; Zubler, N. S.; Taddio, M. F.; Mu, L.; Altmann, K. H.; Krämer, S. D. *In vitro* and *in vivo* evaluation of ABCG2 (BCRP) inhibitors derived from Ko143. *J. Med. Chem.* **2023,** *66* (10), 6782-6797.
6. Braconi, L.; Dei, S.; Contino, M.; Riganti, C.; Bartolucci, G.; Manetti, D.; Romanelli, M. N.; Perrone, M. G.; Colabufo, N. A.; Guglielmo, S.; Teodori, E. Tetrazole and oxadiazole derivatives as bioisosteres of tariquidar and elacridar: new potent P-gp modulators acting as MDR reversers. *Eur. J. Med. Chem.* **2023,** *259*, 115716.
7. Seward, D. J.; Koh, A. S.; Boyer, J. L.; Ballatori, N. Functional complementation between a novel mammalian polygenic transport complex and an evolutionarily ancient organic solute transporter, *OSTα-OSTβ*. *J. Biol. Chem.* **2003,** *278* (30), 27473-27482.
8. Malinen, M. M.; Ali, I.; Bezençon, J.; Beaudoin, J. J.; Brouwer, K. L. R. Organic solute transporter OSTα/β is overexpressed in nonalcoholic steatohepatitis and modulated by drugs associated with liver injury. *Am. J. Physiol. Gastrointest. Liver Physiol.* **2018,** *314* (5), G597-G609.
9. Visser, F.; Vickers, M. F.; Ng, A. M.; Baldwin, S. A.; Young, J. D.; Cass, C. E. Mutation of residue 33 of human equilibrative nucleoside transporters 1 and 2 alters sensitivity to inhibition of transport by dilazep and dipyridamole. *J. Biol. Chem.* **2002,** *277* (1), 395-401.
10. Cabrera, M.; Cui, L. *In vitro* activities of primaquine-schizonticide combinations on asexual blood stages and gametocytes of *Plasmodium falciparum*. *Antimicrob. Agents Chemother.* **2015,** *59* (12), 7650-7656.
11. Muscella, A.; Vetrugno, C.; Biagioni, F.; Calabriso, N.; Calierno, M. T.; Fornai, F.; De Pascali, S. A.; Marsigliante, S.; Fanizzi, F. P. Antitumour and antiangiogenic activities of [Pt(O,O'-acac)(γ-acac)(DMS)] in a xenograft model of human renal cell carcinoma. *Br. J. Pharmacol.* **2016,** *173* (17), 2633-2644.
12. Salehi, R.; Abyar, S.; Ramazani, F.; Khandar, A. A.; Hosseini-Yazdi, S. A.; White, J. M.; Edalati, M.; Kahroba, H.; Talebi, M. Enhanced anticancer potency with reduced nephrotoxicity of newly synthesized platin-based complexes compared with cisplatin. *Sci. Rep.* **2022,** *12* (1), 8316.
13. Descôteaux, C.; Provencher-Mandeville, J.; Mathieu, I.; Perron, V.; Mandal, S. K.; Asselin, E.; Bérubé, G. Synthesis of 17β-estradiol platinum(II) complexes: biological evaluation on breast cancer cell lines. *Bioorg. Med. Chem. Lett.* **2003,** *13* (22), 3927-3931.
14. Ferreira, C. G.; Span, S. W.; Peters, G. J.; Kruyt, F. A.; Giaccone, G. Chemotherapy triggers apoptosis in a caspase-8-dependent and mitochondria-controlled manner in the non-small cell lung cancer cell line NCI-H460. *Cancer Res.* **2000,** *60* (24), 7133-7141.
15. Teixeira, L. J.; Seabra, M.; Reis, E.; da Cruz, M. T.; de Lima, M. C.; Pereira, E.; Miranda, M. A.; Marques, M. P. Cytotoxic activity of metal complexes of biogenic polyamines: polynuclear platinum(II) chelates. *J. Med. Chem.* **2004,** *47* (11), 2917-2925.
16. Thati, B.; Noble, A.; Creaven, B. S.; Walsh, M.; McCann, M.; Kavanagh, K.; Devereux, M.; Egan, D. A. In vitro anti-tumour and cyto-selective effects of coumarin-3-carboxylic acid and three of its hydroxylated derivatives, along with their silver-based complexes, using human epithelial carcinoma cell lines. *Cancer Lett.* **2007,** *248* (2), 321-331.
17. Wilson, S. C.; Howard, P. W.; Forrow, S. M.; Hartley, J. A.; Adams, L. J.; Jenkins, T. C.; Kelland, L. R.; Thurston, D. E. Design, synthesis, and evaluation of a novel sequence-selective epoxide-containing DNA cross-linking agent based on the pyrrolo[2, 1-*c*][1,4]benzodiazepine system. *J. Med. Chem.* **1999,** *42* (20), 4028-4041.

18. Buckley, R. G.; Elsome, A. M.; Fricker, S. P.; Henderson, G. R.; Theobald, B. R.; Parish, R. V.; Howe, B. P.; Kelland, L. R. Antitumor properties of some 2-[(dimethylamino)methyl]phenylgold(III) complexes. *J. Med. Chem.* **1996,** *39* (26), 5208-5214.
19. Hotze, A. C.; Bacac, M.; Velders, A. H.; Jansen, B. A.; Kooijman, H.; Spek, A. L.; Haasnoot, J. G.; Reedijk, J. New cytotoxic and water-soluble bis(2-phenylazopyridine)ruthenium(II) complexes. *J. Med. Chem.* **2003,** *46* (9), 1743-1750.
20. Nayadu, S.; Behera, D.; Sharma, M.; Kaur, G.; Gudi, G. Fluorescent probe based CYP inhibition assay: a high throughput tool for early drug discovery screening. *Int. J. Pharm. Pharm. Sci.* **2013,** *5* (2), 303-307.
21. Donato, M. T.; Jiménez, N.; Castell, J. V.; Gómez-Lechón, M. J. Fluorescence-based assays for screening nine cytochrome P450 (P450) activities in intact cells expressing individual human P450 enzymes. *Drug Metab. Dispos.* **2004,** *32* (7), 699-706.
22. Dehring, K. A.; Workman, H. L.; Miller, K. D.; Mandagere, A.; Poole, S. K. Automated robotic liquid handling/laser-based nephelometry system for high throughput measurement of kinetic aqueous solubility. *J. Pharm. Biomed. Anal.* **2004,** *36* (3), 447-456.
23. McGinnity, D. F.; Soars, M. G.; Urbanowicz, R. A.; Riley, R. J. Evaluation of fresh and cryopreserved hepatocytes as in vitro drug metabolism tools for the prediction of metabolic clearance. *Drug Metab. Dispos.* **2004,** *32* (11), 1247-1253.
24. Lin, X.; Skolnik, S.; Chen, X.; Wang, J. Attenuation of intestinal absorption by major efflux transporters: quantitative tools and strategies using a Caco-2 model. *Drug Metab. Dispos.* **2011,** *39* (2), 265-274.
25. Rolsted, K.; Rapin, N.; Steffansen, B. Simulating kinetic parameters in transporter mediated permeability across Caco-2 cells. A case study of estrone-3-sulfate. *Eur. J. Pharm. Sci.* **2011,** *44* (3), 218-226.
26. Román-Álamo, L.; Avalos-Padilla, Y.; Bouzón-Arnáiz, I.; Iglesias, V.; Fernández-Lajo, J.; Monteiro, J. M.; Rivas, L.; Fisa, R.; Riera, C.; Andreu, D.; Pintado-Grima, C.; Ventura, S.; Arce, E. M.; Muñoz-Torrero, D.; Fernàndez-Busquets, X. Effect of the aggregated protein dye YAT2150 on *Leishmania* parasite viability. *Antimicrob. Agents Chemother.* **2024,** *68* (3), e0112723.
